# Voice Disorder Classification Based on Multitaper Mel Frequency Cepstral Coefficients Features

**DOI:** 10.1155/2015/956249

**Published:** 2015-11-22

**Authors:** Ömer Eskidere, Ahmet Gürhanlı

**Affiliations:** ^1^Department of Electrical Electronics Engineering, Bursa Orhangazi University, 16310 Bursa, Turkey; ^2^Department of Computer Engineering, Bursa Orhangazi University, 16310 Bursa, Turkey

## Abstract

The Mel Frequency Cepstral Coefficients (MFCCs) are widely used in order to extract essential information from a voice signal and became a popular feature extractor used in audio processing. However, MFCC features are usually calculated from a single window (taper) characterized by large variance. This study shows investigations on reducing variance for the classification of two different voice qualities (normal voice and disordered voice) using multitaper MFCC features. We also compare their performance by newly proposed windowing techniques and conventional single-taper technique. The results demonstrate that adapted weighted Thomson multitaper method could distinguish between normal voice and disordered voice better than the results done by the conventional single-taper (Hamming window) technique and two newly proposed windowing methods. The multitaper MFCC features may be helpful in identifying voices at risk for a real pathology that has to be proven later.

## 1. Introduction

Disordered voice quality could be a symptom of a disease related to laryngeal disorders. In clinical practice, the primary approach to assess voice quality is the auditory-perceptual evaluation. For this approach, the severity (degree) and quality of dysphonia are evaluated by a tool such as GRBAS (Grade, Roughness, Breathiness, Asthenia, and Strain) scale [1]. Auditory-perceptual evaluation offers a standardized procedure for assessment of abnormal voice quality. For this approach, voice evaluation is performed subjectively by the clinician's direct audition. Auditory-perceptual evaluation of voice quality is subjective because of the variability between listeners [2]. Moreover, this subjective evaluation can cause inconsistency on judging pathological voice quality [3]. Alternatively, laryngoscopic techniques such as direct laryngoscopy, indirect laryngoscopy, and telescopic video laryngoscopy are invasive tools which allow the observation of vocal folds [4]. These techniques, which are commonly used for monitoring the larynx, make the diagnosis of many laryngeal disorders possible [1]. On the other hand, these monitoring techniques may cause discomfort to the patient and become costly [5].

Apart from the above-mentioned methods, acoustic analysis of voice samples is generally applied as a complementary technique to aid ear, nose, and throat clinicians [6–10]. This analysis technique is an effective and noninvasive approach for the assessment of voice quality. For clinical application, acoustic analysis of disordered voices enables doctors to document quantitatively the degree of different voice qualities and the automatic screening of voice disorders. This technique can also be performed for the evaluation of surgical and pharmacological treatments and rehabilitation processes such as monitoring the patient's progress over the course of voice therapy [11, 12]. Furthermore, in voice clinics, various commercial acoustic analysis computer programs are run to aid the clinician in rating voice quality [13, 14]. Apparently, clinicians and speech therapists commonly combine auditory-perceptual evaluation techniques, laryngoscopic techniques, and acoustic analysis methods to evaluate voice quality.

Recently, many researchers have been working on differentiating between two levels of voice quality, normal and pathological, using acoustic analysis methods [3, 15, 16]. For this aim, the raw voice samples are converted into features which have more useful and compact representations of voice. In the literature, the features such as measures of acoustic perturbation (jitter and shimmer), the harmonics to noise ratio, and the glottal to noise excitation ratio have been applied for assessment of vocal quality [17, 18]. Moreover, nonlinear dynamic methods, including Lyapunov exponents and correlation dimension, have been applied to various kinds of classification tasks for disordered voice samples [19–21]. The recent studies show that these nonlinear methods may be more appropriate for aperiodic voices than traditional perturbation methods [6]. On the other hand, in comparison with perturbation analysis, the drawback of these nonlinear methods is the fact that they are more complex and may need longer computation time [22].

The well-known MFCC feature extraction has been commonly used in automatic classification between healthy and impaired voices [15]. This technique can be considered as an approach of the structure of human auditory perception [23]. Usually MFCC parameters are computed from a windowed periodogram using short time frames of speech via discrete Fourier transform algorithm. In this case, windowing attempts to reduce bias but large variance is still a problem. The large variance for spectrum estimation can be reduced by replacing the Hamming-windowed power spectrum with multiple time domain windows. This is usually called the multitaper spectral estimation method [24–26]. The idea in the multitaper spectral estimation method is to analyze the speech frame using a number *N* of spectrum estimators, each having a different taper, and then to compute the final spectrum as a weighted mean of each subspectrum. In [25], it is shown that multiple window spectral estimates have smaller variance than single windowed spectrum estimates by a factor that approaches 1/*N*.

For a long time, multitaper spectrum estimation has been used in geographical applications [27] and has demonstrated good results. But little attention has been paid to multitaper spectrum estimation in the field of speech processing. Recently researchers have started to employ the method in speech processing as well [24]. This study demonstrated first time that multitaper MFCC features could be used for speaker verification systems. Then, this method was applied to the speech recognition [28], emotion recognition [29], and language identification [30] and was shown to result in better performance than the single windowed method. In this study, our goal is to investigate the usage of multitaper MFCC features in the automatic discrimination of two levels of voice quality (healthy and pathological voices). So as to evaluate the usefulness of the proposed method, an automatic classification system is employed. To our knowledge, there were no previous studies in the existing literature using multitaper MFCC features for this problem. The second objective of this study is to apply different multitaper techniques including multipeak method [31], SWCE (sinusoidal weighted cepstrum estimator) method [32], and Thomson method [33] to MFCC and compare their performance to novel proposed windowing techniques [34, 35] and single-taper technique. In addition, the number of tapers affecting the classification performance and the issues of weight selection in the Thomson method are investigated. Experimental results indicate that, with a suitable configuration, the multitaper method outperformed these windowing techniques.

The outline of the paper is as follows. Multitaper spectrum estimation method and novel windowing techniques are given in Section 2.  Section 3 evaluates the efficiency of the multitaper spectrum estimation for the classification of voice qualities. Discussion is presented in Section 4 and then conclusion is given in Section 5.

## 2. Methods

### 2.1. Multitaper Spectrum Estimation

In MFCC feature extraction process, the power spectrum is computed from a windowed periodogram. The short-time power spectrum estimate $\hat{S}\left(f\right)$ is given by

(1) $$\begin{array}{c} \hat{S}\left(\;f\;\right)={\left|\;\overset{L-1}{\underset{t=0}{\sum }}w\left(\;t\;\right)x\left(\;t\;\right)e^{-2i\pi f/L}\;\right|}^{2}, \end{array}$$

where *x* = [*x*(0), *x*(1), *x*(2),…, *x*(*L* − 1)] is a frame of utterance with length *L*, *f* ∈ {0,1, 2,…, *L* − 1} is frequency bin index, *i* is the imaginary unit, and *w*(*t*) denotes a window function. For MFCC application,* Hamming* window is the most popular window and we choose this window; it is given by

(2) $$\begin{array}{c} w\left(\;t\;\right)=0.54-0.46\cos\left(\;\frac{2\pi t}{L}\;\right). \end{array}$$

**Figure 1:**
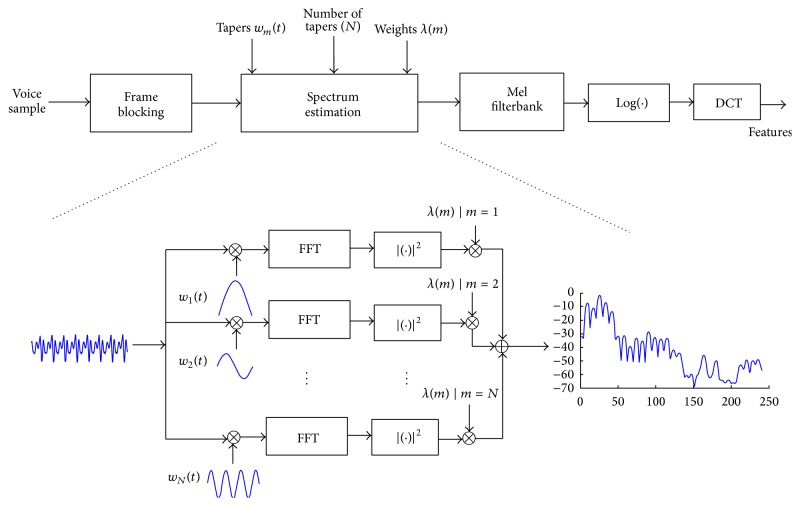
Block diagram of single-taper and multitaper spectrum estimation based on MFCC feature extraction.

A single taper (e.g., Hamming window) reduces the bias of the spectrum which is the difference between the estimated spectrum $\hat{S}\left(f\right)$ and the actual spectrum *S*(*f*) but the estimated spectrum has higher variance. This problem can be reduced by multitaper spectrum estimator [25]. The multitaper spectrum estimator can be expressed as

(3) $$\begin{array}{c} {\hat{S}}_{MT}\left(\;f\;\right)=\overset{N}{\underset{m=1}{\sum }}\lambda \left(\;m\;\right){\left|\;\overset{L-1}{\underset{t=0}{\sum }}w_{m}\left(\;t\;\right)x\left(\;t\;\right)e^{-2i\pi f/L}\;\right|}^{2}, \end{array}$$

where *N* is the number of the tapers, *w*_*m*_ is the *m*th data taper (*m* = 1,2,…, *N*), and *λ*(*m*) is the weight of the *m*th taper. In this method, spectrum estimation is obtained from a series of spectra which are weighted and averaged in frequency domain. The block diagram of MFCC extraction from the single-taper and multitaper spectrum estimation is presented in Figure 1. As a special case, if *m* = *N* = 1 and *λ*(*m*) = 1, (3) simply degrades to (1) and in this case a single windowed power spectrum is obtained.

**Figure 2:**
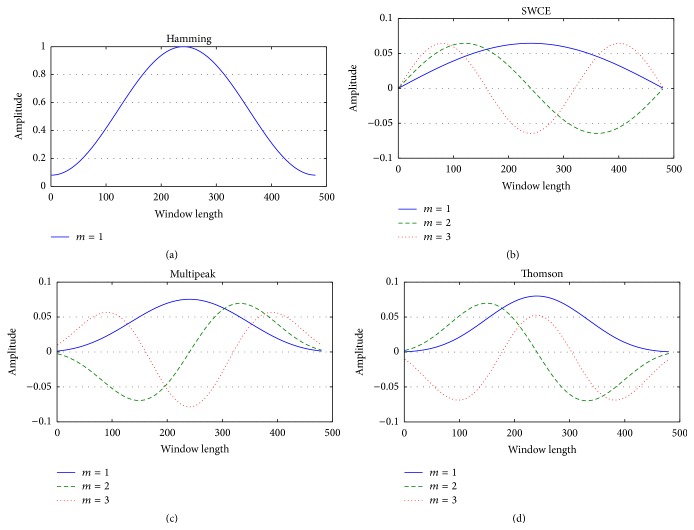
Single taper and different multitapers used for spectrum estimation: (a) Hamming window, (b) the sine tapers, (c) the multipeak tapers, and (d) the Thomson tapers. Window length is 480; *m* is the taper number.

Some of the multitaper methods in the literature are Thomson multitaper, multipeak multitaper, and SWCE (sinusoidal weighted cepstrum estimator) multitaper, which are based on the Slepian tapers [17], peak matched multiple tapers, and sine tapers, respectively. These multitapers and Hamming taper are demonstrated in Figure 2. One goal of this study is to evaluate the effect of these tapers and compare their performances for a voice disorder classification system. Details of these tapers may be found in [31–33].

**Figure 3:**
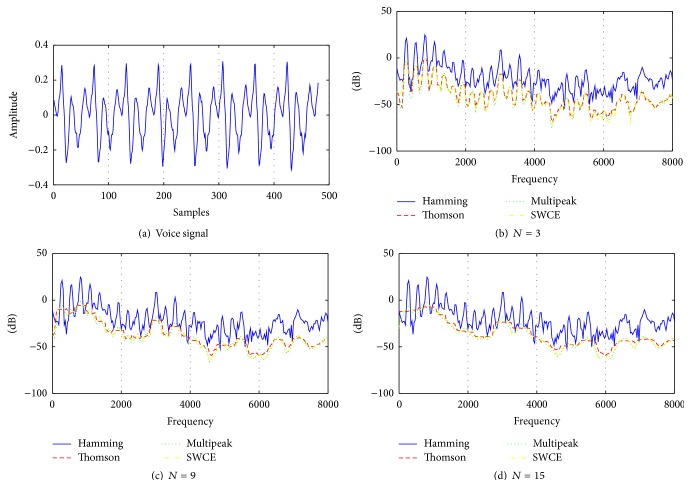
(a) Normal voice and (b), (c), and (d) its estimated spectrum by the single taper (Hamming) and Thomson, multipeak, and SWCE multitaper methods for *N* = 3 tapers, for *N* = 9 tapers, and for *N* = 15 tapers, respectively.

**Figure 4:**
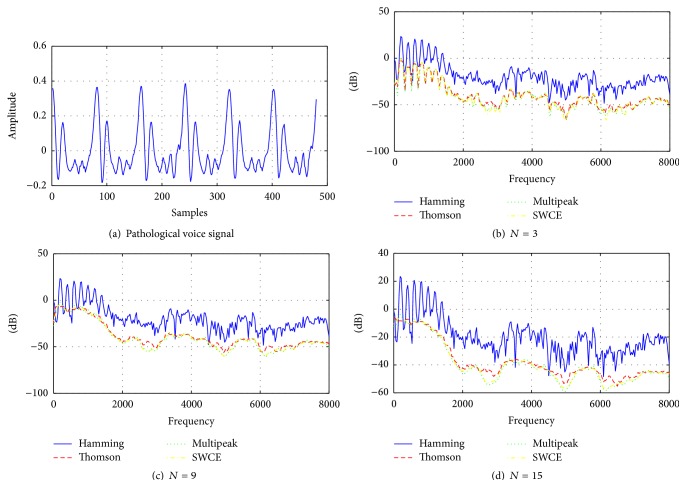
(a) Pathological voice and (b), (c), and (d) its estimated spectrum by the single taper (Hamming) and Thomson, multipeak, and SWCE multitaper methods for *N* = 3 tapers, for *N* = 9 tapers, and for *N* = 15 tapers, respectively.

To make a visual comparison, samples from normal and pathologically affected voices for vowel /a/ and their estimated spectra by the Hamming windowed DFT spectrum as a reference and Thomson, multipeak, and SWCE multitaper methods are given in Figures 3 and 4. The number of tapers used for the multitaper methods is 3, 9, and 15, with a frame length of 30 msec and the sampling frequency is 16 kHz.

In Figures 3 and 4, it is shown that each multitaper method has a different spectrum. For the same value of *N*, multipeak spectrum estimation has sharper peaks than Thomson and SWCE methods. Additionally, the single-taper spectrum includes more details comparing it with these multitaper methods and it can be expected that this multitaper spectral estimation has smaller variance. As these techniques generate different spectrum on the same voice frame, the results cause different cepstrum coefficients [25].

In estimating the spectrum by multitapering, the first taper attributes more weight to the center of the short-term signal than to its ends, while higher order tapers attribute increasingly more weight to the ends of the frame. For the SWCE multitaper method weights can be found from

(4) λm=cos⁡2πm−1/N/2+1∑m=1Ncos⁡2πm−1/N/2+1,m=1,2,…,N.

Multipeak multitaper method weights can be defined as

(5) $$\begin{array}{c} \lambda \left(\;m\;\right)=\frac{v_{m}}{\sum_{m=1}^{N}v_{m}},\;\left(m=1,2,\ldots ,N\right), \end{array}$$

where *v*_*m*_ is the eigenvalues of the multiple windows.

**Figure 5:**
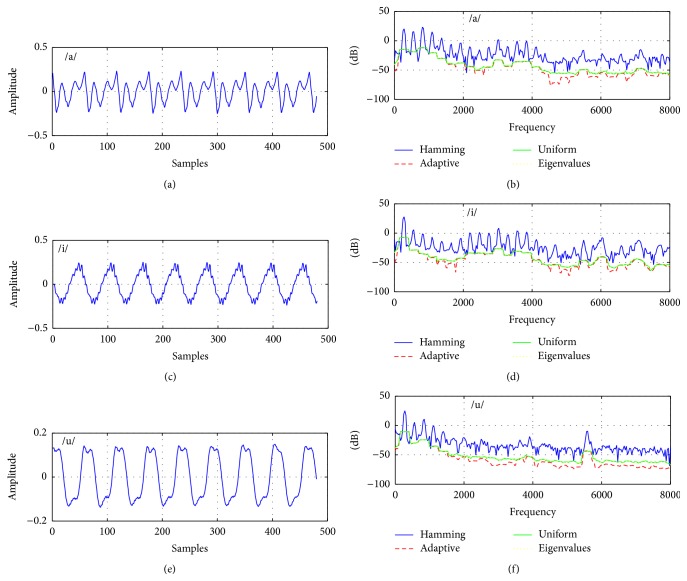
(a), (c), and (e) Sustained vowels /a/, /i/, and /u/ from normal subjects and (b), (d), and (f) their Thomson multitaper spectral estimates using uniform weights, eigenvalues as the weights, and adaptive weights.

**Figure 6:**
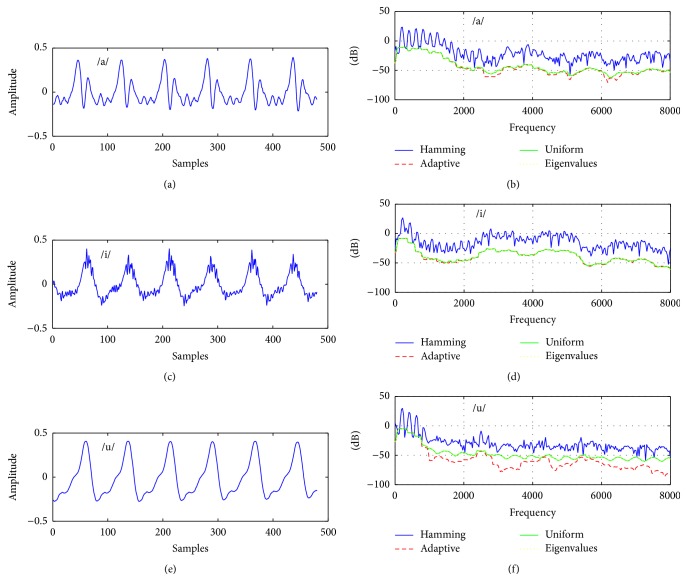
(a), (c), and (e) Sustained vowels /a/, /i/, and /u/ from pathological subjects and (b), (d), and (f) their Thomson multitaper spectral estimates using uniform weights, eigenvalues as the weights, and adaptive weights.

Usually, the three different approaches can be used for weighting schemes in the Thomson multitaper. These are uniform weights, where *λ*(*m*) = 1/*N*  (*N* is the number of the Slepian tapers), eigenvalue weights, where *λ*(*m*) = *v* ^*m*^ (*v* is the eigenvalues of the Slepian tapers), and adaptive weights, where (*m*) = 1/∑_*i*=1_^*m*^ *v* ^*i*^. Figures 5 and 6 show a comparison of these weighting schemes used in the Thomson multitaper for normal and pathological voice samples (/a/, /i/, and /u/). In speaker verification experiments, uniform weights are used to obtain MFCC multitaper features [24–26]. In [36], adaptive weights give higher accuracy than the uniform and eigenvalue weighting schemes. Therefore, it may not be clear which weighting technique in the Thomson multitaper is suitable for modeling voice signal. For this reason, we also investigated optimum weighting techniques in the Thomson multitaper for voice disorder classification task.

### 2.2. The Novel Window Methods

Recently, apart from the multitaper method, the novel windowing techniques are presented for signal analysis. In 2011, Mottaghi-Kashtiban and Shayesteh [34] proposed a new efficient window function and compared main lobe width and peak side lobe amplitude to the Hamming window. The proposed window function can be expressed as

(6) $$\begin{array}{c} w_{k}\left(\;t\;\right)=a_{0}-a_{1}\cos\left(\;\frac{2\pi t}{L}\;\right)-a_{3}\cos\left(\;\frac{6\pi t}{L}\;\right), \end{array}$$

where *a*_0_ = 0.5363 − 0.14/*L*, *a*_1_ = 0.996 − *a*_0_, and *a*_3_ = 0.04. This new window function was obtained by the third harmonic of the cosine function in (2). Also they found the suitable amplitudes of DC term to minimize the peak side lobe amplitude [34].

In 2013, Sahidullah and Saha [35] presented a novel family of windowing method to calculate MFCC features. The basic idea of the proposed method is to use a simple time domain processing of signal after it is multiplied with a single window. The new window function can be expressed as

(7) $$\begin{array}{c} w_{s}\left(\;t\;\right)=t^{\tau }w\left(\;t\;\right),\;\tau =1,2,\ldots . \end{array}$$

**Figure 7:**
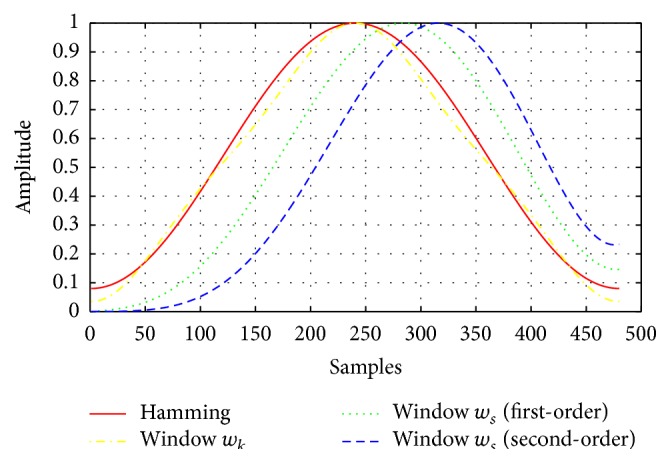
The two novel window functions and Hamming window in the time domain.

In the case where *τ* = 0, the window function is equal to *w*(*t*) such as Hamming window.  Figure 7 shows these novel windowing functions and Hamming window as a reference in the time domain. For window *w*_*s*_, first-order and second-order (*τ* = 1 and *τ* = 2) window functions are used and amplitude of all the windows is normalized to 1 for visual clarity. In this study, we investigate the effects of these windowing techniques and compared them to the multitaper methods to categorize normal voice quality from disordered voice quality.

## 3. Experiments

The performance of the proposed multitaper MFCC features is evaluated on an open database, namely, Saarbruecken Voice (SV) database, developed by Putzer [37, 38]. This database consists of pathological and healthy voices at different pitches (low, normal, and high) from more than 2000 speakers. SV database includes simultaneous voice and electroglottography (EGG) recordings of sustained vowels /a/, /i/, and /u/ for each case. The files have averages of around 1 and 3 s for sustained vowels and voice samples were sampled at 50 kHz with 16 bits of resolution.

In this study, voice samples of sustained vowels /a/, /i/, and /u/ produced at the subjects' normal pitch were used from SV database. Each voice signal resampled at 16 kHz was considered. For this work, 650 normal subjects and a group of 650 subjects with functional and organic dysphonia voice pathologies were chosen from SV database. The details of voice samples used in the study can be seen in Table 1.

In the experiments, the voice samples were segmented into frames of 30 ms lengths and the frame shift is 15 ms. Afterwards, each frame was weighted by a single window or multitaper method. To generate SWCE, multipeak, and Thomson tapers, the multitaper functions were utilized as described by Kinnunen et al. [25]. Afterwards, 29-channel Mel frequency filter bank was applied on the short-time spectrum. Then, the logarithmically compressed filter bank outputs were calculated and the DCT was applied on the filter bank outputs. The first 12 cepstral coefficients were taken as features excluding energy coefficient *c*_0_ and these features were normalized to the range of 0-1.

For evaluation, we have used Gaussian Mixture Model (GMM) to represent each class. In this approach, voice samples were modeled as a weighted sum of multivariate Gaussian probability density functions. In the GMM parameter estimation, the distribution of features is modeled by the mean vectors ${\vec{\mu }}_{i}$, covariance matrices ∑_*i*_, and mixture weights *c*_*i*_ which is denoted by the notation Θ = {*c*_*i*_, *μ*_*i*_, ∑_*i*_}, *i* = 1,2,…, *K*, where *K* is the number of mixture components [39]. These model parameters (Θ) are commonly determined using expectation maximization (EM) algorithm. Finding these parameters, this procedure iteratively updates the parameters by maximizing the expected log-likelihood of the data, and it guarantees a monotonic increase in the model's log-likelihood value [40, 41]. The classification of a sequence test feature vector is based on the calculation of a simple set of likelihood functions using the test voice. In other words, a test frame is classified with a normal or pathological class label, the result of which is the largest likelihood function, indicating the most likely class. In the proposed system, we have used 16 mixture components with diagonal covariance matrices for GMM classifier. We have used half of the features for training and the rest for testing randomly and all the experiments are repeated 20 times. Finally, the system performance was computed by averaging the results obtained from each experiment.

## 4. Results

**Figure 8:**
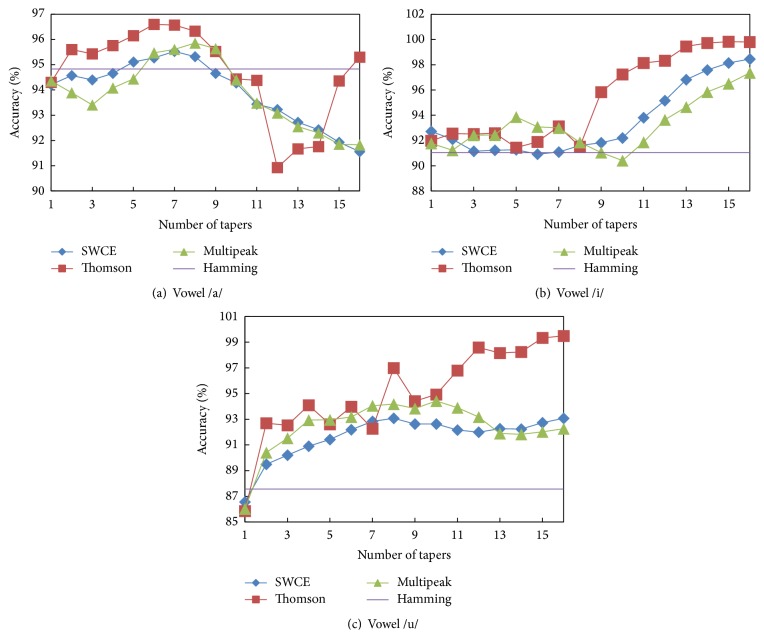
Classification accuracies (%) using different number of tapers for (a) sustained vowel /a/, (b) sustained vowel /i/, and (c) sustained vowel /u/.

We first evaluated the multitaper spectrum estimation technique described in Figure 1 for different numbers of tapers. In the previous multitaper applications, different numbers of tapers were applied to speech recognition [28] and speaker verification problems [24, 25, 36]. The dataset that was used previously is different from the voice quality classification experiments. Therefore, the previous conclusion that the optimal number of tapers, *N*, was found from 4 to 8 is no longer suitable to our task. For sustained vowels /a/, /i/, and /u/, the best value of *N* in our case should be rediscovered. Moreover, we compare the classification accuracies of the SWCE, Thomson (using uniform weights), and multipeak systems and illustrate the conventional Hamming windowing method as a reference in Figure 8.

In Figure 8, it can be seen that the multitaper methods outperform the baseline Hamming method depending on the number of tapers. In the case of vowel /a/, the Thomson multitaper method performs relatively better for 6 ≤ *N* ≤ 8 taper values than the other methods. For /i/ and /u/ vowels, it is observed that the multitaper methods outperform Hamming method in nearly all cases and this is because the exact setting is not very critical for these vowels.

**Figure 9:**
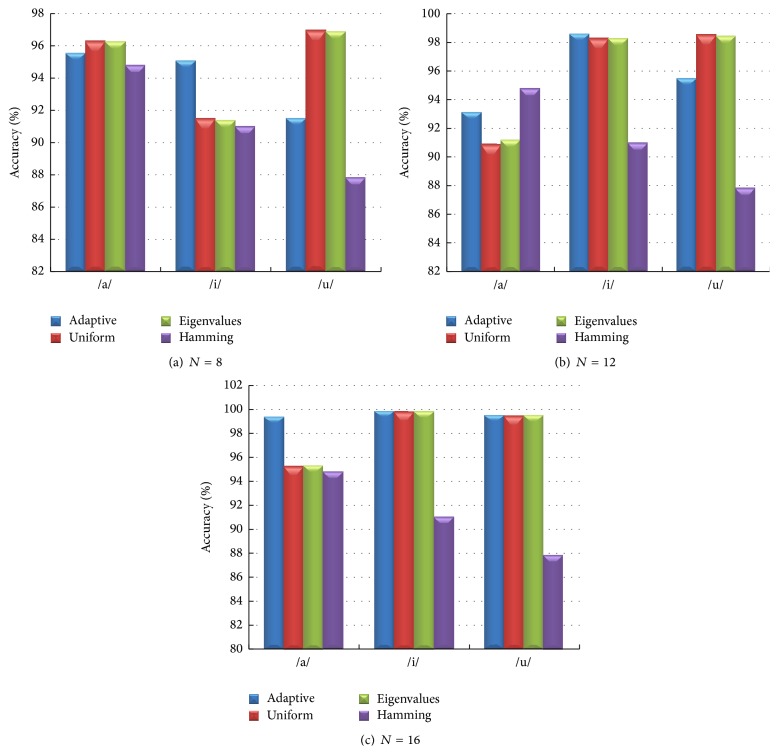
Voice quality classification accuracies (for /a/, /i/, and /u/) using the weights of Thomson multitaper method and Hamming window with (a)  *N* = 8, (b)  *N* = 12, and (c)  *N* = 16.

We next compared the weights of the Thomson multitaper: uniform, eigenvalue, and adaptive weights. In the experiments, we use the number of tapers as *N* = 8, 12, and 16 for each multitaper method, respectively. The classification performance results are demonstrated in Figure 9.

When comparing the performances of the weights of the Thomson multitaper method, all three weighting techniques outperformed the baseline Hamming method. For vowels /a/, /i/, and /u/, the highest accuracies are obtained using *N* = 16 with adaptive weights.

**Figure 10:**
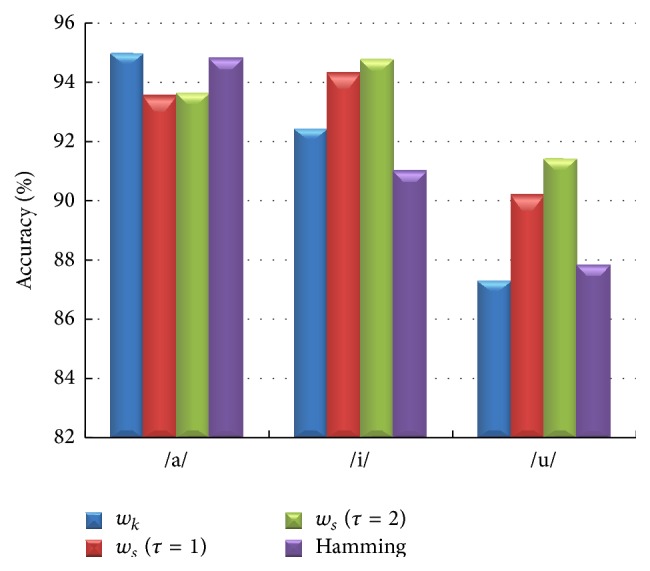
Classification performance comparisons of the two different window functions and Hamming window for /a/, /i/, and /u/ vowels.

Additionally, the classification task applied to the novel proposed weighting schemas in [34, 35] compared with baseline Hamming method offers interesting results. As shown in Figure 10, our classification experiment on SV database yields the highest accuracies of 95% (vowel /a/) for window *w*_*k*_ system and 94.78% (vowel /i/) and 91.42% (vowel /u/) using window *w*_*s*_  (*τ* = 2) system.

Table 2 summarizes the classification results of all windowing methods and the multitaper systems. The baseline results on the test set were obtained by using Hamming windowed MFCCs on the vowels /a/, /i/, and /u/. In the multitaper experiments, the number of tapers was set to 16 and adaptive weights were used in the Thomson method. Additionally, we fix *τ* = 2 for window *w*_*s*_.

As seen in Table 2, Thomson multitaper method with adaptive weighting was observed as the highest accuracy improvement of 4.8% for vowel /a/, 9.7% for vowel /i/, and 13.29% for vowel /u/, respectively. When comparing all multitaper methods together over the baseline, we observe that the Thomson method is preferable.

## 5. Discussion

In this paper, we have compared the performance of different windowing techniques using MFCC in order to investigate how to discriminate voice disorders from healthy controls. This classification problem has attracted interest in recent years, with the best results reporting approximately 79% recognition accuracy [42] on SV database. In [38], 76.4% accuracy was obtained using a new parameterization of voice quality properties in the voice signal. Here, we indicated that we can achieve almost 99% accuracy using multitaper MFCCs. Compared to previous studies in this application, we have used recently proposed windowing techniques and multitaper spectrum estimation methods which have not been previously used in voice quality classification task.

Moreover, we discussed the effect of chosen multitaper parameters such as the number of tapers, type of taper, and the weights of the Thomson multitaper method. In this work, the optimum number of tapers is 6 for vowel /a/, 15 for vowel /i/, and 16 for vowel /u/ (see Figure 8). The optimum number of tapers changes application and dataset [25–30]. In [24], the bias, variance, and MSE (squared bias plus variance) of the MFCC estimator were investigated using a set of 50 different recordings of the phonemes /a/ and /l/. Sandberget al. found that multitapers (multipeak, SWCE, and Thomson) with *N* between 8 and 16 indicate a good tradeoff between bias and variance for most MFCCs. In this paper, we obtained similar results using multitaper MFCCs for voice quality classification issues and it is clearly seen that the number of tapers is an important parameter. Moreover, the optimum weight of the Thomson multitaper method was found to be adaptive weights for the phonemes /a/, /i/, and /u/.

As can be seen from Table 2, the proposed multitaper method provides better classification results than other newly proposed windowing methods in [34, 35] and popular Hamming window. For voice quality classification problems, it is found that the Thomson multitaper method can be chosen as the optimal tapering method which is designed for smooth spectrum especially white noise [24]. This is expected because the disordered voice samples contain more noise compared to the healthy voices and the spectrum of these voice samples is estimated better by using the multitaper method than by using the single-taper method. In other words, the single-taper spectrum comprises more details for a voice frame, while the multitaper spectra contain a smoother voice frame and this situation can be seen in Figures 3 and 4. Thus, averaging spectral estimates with this method helps to reduce large variance especially for the Thomson multitaper method (see Figures 5 and 6) comparable to the single-tapered spectrum estimate. For this reason, in differentiating pathological voices from the healthy ones, multitaper MFCCs give better performance.

## 6. Conclusion

In the present study, we have investigated multitaper MFCC systems for a voice quality classification task. The Thomson, SWCE, and multipeak MFCC systems and GMM based modeling techniques were employed for this task. The system was tested using sustained vowels (/a/, /i/, and /u/) from 650 normal and 650 pathological subjects. The experimental results showed that the Thomson method (using adaptive weights and *N* = 16) outperformed the SWCE and multipeak MFCC systems as well as the baseline Hamming window system. Moreover, it was found that the important parameters such as the number of tapers used for the multitaper methods and the type of the weights in the Thomson method could affect the voice quality classification performance. Furthermore, it was found that the multitaper based features performed slightly better in terms of accuracy than the novel proposed windowing based features in most cases. These results confirm that multitaper methods (specifically the adaptive weighted Thomson multitaper MFCC) can be an alternative to the traditional MFCC which uses the Hamming window for automatic classification of voice quality. As a result, acoustic assessment techniques (e.g., multitaper MFCC) by no means need to replace auditory-perceptual techniques or laryngoscopic techniques, but they could help improve the voice quality analysis tools available to the clinician.

## Figures and Tables

**Table 1:** Details of voice samples used in the study.

Diagnosis	Number of samples
Cyst	6
Functional dysphonia	76
Hyperfunctional dysphonia	68
Hypofunctional dysphonia	16
Laryngitis	102
Leukoplakia	41
Normal	650
Paralysis	196
Reinke's edema	66
Vocal fold cancer	22
Vocal fold polyp	41
Vocal nodule	16

**Table 2:** Average relative improvement in SV database obtained by the novel window functions and the multitaper systems over the baseline Hamming window system.

Vowel	Baseline acc. (%)	Method	Acc. (%)	Impr. (%)
/a/	94.83	Window *w*_*k*_	95.00	0.18
		Window *w*_*s*_	93.65	−1.24
		SWCE	91.56	−3.45
		Multipeak	91.83	−3.16
		Thomson	99.38	4.8

/i/	91.03	Window *w*_*k*_	92.45	1.56
		Window *w*_*s*_	94.78	4.12
		SWCE	98.45	8.15
		Multipeak	97.37	6.96
		Thomson	99.86	9.7

/u/	87.86	Window *w*_*k*_	87.31	−0.63
		Window *w*_*s*_	91.42	4.05
		SWCE	93.08	5.94
		Multipeak	92.26	5.01
		Thomson	99.54	13.29

Acc., accuracy; Impr., improvement; window *w*_*k*_, a new window function proposed in [34]; window *w*_*s*_, a new window function proposed in [35]; SWCE, sinusoidal weighted cepstrum estimator.
